# Temporal variations in methane emissions from emergent aquatic macrophytes in two boreonemoral lakes

**DOI:** 10.1093/aobpla/plx029

**Published:** 2017-07-04

**Authors:** Per Milberg, Lina Törnqvist, Lars M. Westerberg, David Bastviken

**Affiliations:** 1 Department of Physics, Chemistry and Biology, Conservation Ecology Group, Linköping University, SE 581 83, Linköping, Sweden; 2 Department of Thematic Studies - Environmental Change, Linköping University, SE 581 83, Linköping, Sweden

**Keywords:** Carex, CH_4_ emission, emergent aquatic macrophytes, lakes, *Phragmites australis*, temporal variability

## Abstract

Methane (CH_4_) emissions via emergent aquatic macrophytes can contribute substantially to the global CH_4_ balance. We addressed temporal variability in CH_4_ flux by using the static chamber approach to quantify fluxes from plots dominated by two species considered to differ in flux transport mechanisms (*Phragmites australis*, *Carex rostrata*). Temporal variability in daily mean emissions from early June to early October was substantial. The variable that best explained this variation was air temperature. Regular and consistent diel changes were absent and therefore less relevant to include when estimating or modelling CH_4_ emissions. Methane emissions per m^2^ from nearby plots were similar for *Phragmites australis* and *Carex rostrata* indicating that CH_4_ production in the system influenced emissions more than the species identity. This study indicates that previously observed diel patterns and species-effects on emissions require further evaluation to support improved local and regional CH_4_ flux assessments.

## Teaser

Aquatic plants can function as conduits for methane from sediment to the air. We studied the flux of methane from plots, in two Swedish lakes, dominated by two species (Phragmites australis, Carex rostrata). There were substantial seasonal differences (June to October), mainly tracking air temperature. There were large differences in flux during 24 h sampling, but with no consistent patterns between days. Emissions per m2 were similar for the two species. This suggests a system where CH4 production shapes emissions while species identity, and diurnal patterns, play a smaller role.

## Introduction

Methane (CH_4_) impacts the global energy balance and climate, has substantially higher global warming potential than carbon dioxide (CO_2_) per kg in a 100-year perspective, and accounts for some 20 % of radiative forcing (Myhre *et al.* 2013). Wetlands and inland waters are large contributors to overall CH_4_ emission (Laanbroek 2010; Bastviken *et al.* 2011; Ciais *et al.* 2013; Kirschke *et al.* 2013), where anaerobic degradation of organic matter produces CH_4_ through methanogenesis. Upon transport from the sediments, a large proportion of dissolved CH_4_ can be oxidized in surface sediments or in the water (Bastviken *et al.* 2008; Conrad 2009; Laanbroek 2010; Duc *et al.* 2010). Therefore, the fluxes of dissolved CH_4_ from surface waters are often smaller than flux pathways by which CH_4_ can ‘escape’ oxidation, such as ebullition (flux by bubbles from sediments) and flux through rooted emergent aquatic macrophytes (Joabsson *et al.* 1999; Bastviken 2009). Such plants have well-developed arenchema in their stems and underground rhizomes that transport oxygen to roots (Laanbroek 2010). These gas transport systems can also transport CH_4_ molecules from the root systems, bypassing the oxidation zone in the sediments and release methane directly into the atmosphere via stomata (Yavitt and Knapp 1998).

Emergent aquatic macrophytes in the arctic wet tundra play an important role in the exchange of CH_4_ between the anaerobic environment and the atmosphere (Joabsson *et al.* 1999; Ding *et al.* 2005; Carmichael *et al.* 2014). It is widely suggested that emergent aquatic macrophytes are key sources of CH_4_ emissions to the atmosphere through the aerenchyma (Ding *et al.* 2005; Bhullar *et al.* 2013). In addition, emergent aquatic macrophytes contribute carbon to the sediment during the growing season, by production of plant biomass and root leakage, further increasing CH_4_ production in wetlands (Ding *et al.* 2005; Laanbroek 2010).

Molecular diffusion and convective flow are common processes that have been proposed to regulate transport of CH_4_ in plant tissues (Kim *et al.* 1998), the latter depending on the pressure gradient in the plant (Tornberg *et al.* 1994). It has been suggested that observed differences between day and night in CH_4_ fluxes may be due to molecular diffusion during night and convective flow during the day (Kim *et al.* 1998). The dominant type of gas transport can vary between species and previous work indicated a strong influence of convective gas flow in *Phragmites australis* (Armstrong and Armstrong 1991, 2014) whereas molecular diffusion through the aerenchyma has been suggested to dominate in *Carex rostrata* (Chanton and Whiting 1996). Therefore, it has been assumed that flux mediated by aquatic plants is highly species-dependent (Joabsson *et al.* 1999).

While plant species and gas transport mechanisms have been seen as important for wetland CH_4_ emissions, fluxes can also be regulated by environmental variables, which can influence plant activity and ecosystem CH_4_ production. For example, air temperature, wind, pressure, humidity and light are factors suggested to control the convective flow (Kim *et al.* 1998). Plant CH_4_ exchange may also be affected by plant growth and amount of active aboveground biomass, for which net ecosystem exchange (NEE; net CO_2_ exchange illustrating the overall balance between the photosynthesis and respiration) can be used as a proxy. The overall potential for CH_4_ emissions is regulated by CH_4_ production in sediments, which also depends on multiple environmental factors such as organic substrate supply and temperature (Yvon-Durocher *et al.* 2014).

Knowing the spatial and temporal variability in methane emission from emergent aquatic macrophytes is essential for assessing their importance, and for monitoring and modelling such emissions. Spatial variability in open water fluxes has recently been shown to depend on distance to inlets and on water depth (e.g. Natchimuthu *et al.* 2016, 2017). For fluxes via emergent aquatic plants, spatial variability has frequently been associated with species distributions (see above), while considerable temporal variability has also been noted. Methane emissions from emergent aquatic macrophytes have been observed to vary over the growing season with a peak in the middle (Hyvönen *et al.* 1998; Juutinen *et al.* 2003; Kankaala *et al.* 2004; Duan *et al.* 2005; Kankaala *et al.* 2005). In lakes in both boreal (Kankaala *et al.* 2005) and arid environments (Duan *et al.* 2005), maximum CH_4_ emission occurred in late June to mid-August, when temperature, growth and biomass peak. Other studies have also shown a positive relationship between CH_4_ emissions and plant biomass during the growing season (Hirota *et al.* 2004; Kankaala *et al.* 2005), but this pattern is not always prominent (Kankaala *et al.* 2004).

There are also reports of considerable diel fluctuations in plant-mediated CH_4_ emissions in the literature (Whiting and Chanton 1996; Käki *et al.* 2001; Juutinen *et al.* 2004; Ding *et al.* 2004; Duan *et al.* 2005). Previous studies have observed that emissions from *Typha latifolia* peak in the morning (Whiting and Chanton 1996), whereas *Peltandra virginica* (Whiting and Chanton 1996) and *Phragmites australis* (Brix *et al.* 1996) peak in the afternoon. Consequently, it has been suggested that diel fluctuations differ among species (Whiting and Chanton 1996). In addition, temperature, light, humidity and other environmental variables may influence diel patterns (Brix *et al.* 1996; Bergström *et al.* 2007; Duan *et al.* 2005; Hirota *et al.* 2004; Juutinen *et al.* 2004; Kankaala *et al.* 2005; Wang and Han 2005). The available studies on diel variations from specific stands of plants are limited as only a few diel cycles have been studied in each environment with relatively few measurements during the 24 h period (Hyvönen *et al.* 1998; Käki *et al.* 2001; Ding *et al.* 2004; Juutinen *et al.* 2004; Xing *et al.* 2004).

Altogether, there are a number of common assumptions about how species versus environmental factors influence fluxes, and about diel flux variability, that are based on limited data, and it is not clear to what extent the prevailing views are valid across ecosystems and over time. Hence, it is important to study diel variability for species with different evolutionary history as well as differences in morphological and anatomical characteristics over whole growth seasons to resolve the influence of environmental variables versus plant species differences and to allow the development of generally valid models.

In the present study, both diel and seasonal variability in CH_4_ emissions from areas dominated by the emergent aquatic macrophytes *Phragmites australis* (Cav.) Steud. and *Carex rostrata* Stokes in two hemiboreal lakes in the southwest of Sweden were measured. The aims were:

- Evaluate diel and seasonal patterns during the same time periods in plant-mediated CH_4_ emissions from emergent aquatic macrophytes species reported to have different dominating flux transport mechanisms (convective or diffusive);
- To evaluate links between CH_4_ fluxes and environmental variables that are potentially related to temporal patterns in plant mediated CH_4_ emissions, such as air temperature, light, air pressure, humidity, NEE of CO_2_, wind, species and biomass.

## Methods

### Study site

The field work was carried out from June to October 2014 in two lakes located in the Skogaryd Research Catchment (www.fieldsites.se (13 July 2017); near Vänersborg, Sweden): Lake Erssjön and Lake Följesjön in the drainage area of Bäveån in Västra Götaland County. Both lakes are located in the hemiboreal zone in the southwest of Sweden. Erssjön (N 58° 22.2786', E 12° 9.7175') is a small open-water lake (6.2 ha) with a mean depth of 1.7 m and a maximum depth of 4.5 m. The vegetation along the lake margin is dominated by *Phragmites australis*, and different species of *Carex*, including *C. rostrata* in the shallower parts, with smaller contributions of *Equisetum fluviatile* and *Typha latifolia*, while *Nymphaea alba* is present at deeper water down to ∼2 m depth. The surrounding area of Erssjön consists of coniferous forest with a small proportion of deciduous trees, as well as agricultural land adjacent to the northern shore of the lake. Följesjön (N 58° 22.5250', E 12° 9.2237') on the other hand, is smaller (3.8 ha), shallower (mean and max depth of 0.5 and 1.5 m, respectively) and almost the entire surface of the lake is dominated by emergent and floating-leaved wetland species. Only small patches of open water remain and even if named as a lake (‘sjön’  =  ‘the lake’ in Swedish), large parts of Följesjön share most characteristics with a plant-covered fen-type wetland. In the middle part of Följesjön, vegetation is dominated by *Phragmites australis*, *Carex* spp. with smaller contributions of *Typha latifolia* while *Sphagnum* spp. and *Myrica gale* are abundant along the shore. The vegetation surrounding Följesjön is similar to the vegetation around Erssjön, except that there is no agricultural land adjacent to Följesjön. The lakes are hydrologically connected, as one of the main streams providing water to Följesjön comes from Erssjön. The distance between the lakes is ∼1 km.

The concentration of P, N and Fe was higher in Följesjön than in Erssjön, whereas mean dissolved O_2_ concentration was lower in Följesjön than in Erssjön (Table 1). Weather variables did not differ considerably between the two lakes.

**Table 1:** Lake characteristics in Lake Erssjön and Lake Följesjön. Wind, temperature and air pressure records from May to October 2014.

Lake	Erssjön		Följesjön	
Area (ha)	6.2		3.8	
Mean depth (m)	1.7		0.5	
	Mean	SD	Mean	SD
tot-P (µg L^−1^)	33.73	10.56	50.77	13.73
tot-N (mg L^−1^)	1.03	0.16	1.43	0.25
tot-Fe (µg L^−1^)	1378	671	1592	500
tot-Mn (µg L^−1^)	135	97	108	47
O_2_ (mg L^−1^)	7.37	1.39	4.72	1.70
Wind (m s^−1^)	1.68	1.01	1.24	0.35
Air temperature (°C)	14.3	5.99	15.18	5.89
Incoming rad (short wave) (W m^−2^)	250	151	246	123
Air pressure (atm)	0.99	0.00	0.99	0.00

### Sampling sites and duration

**Figure 1.**
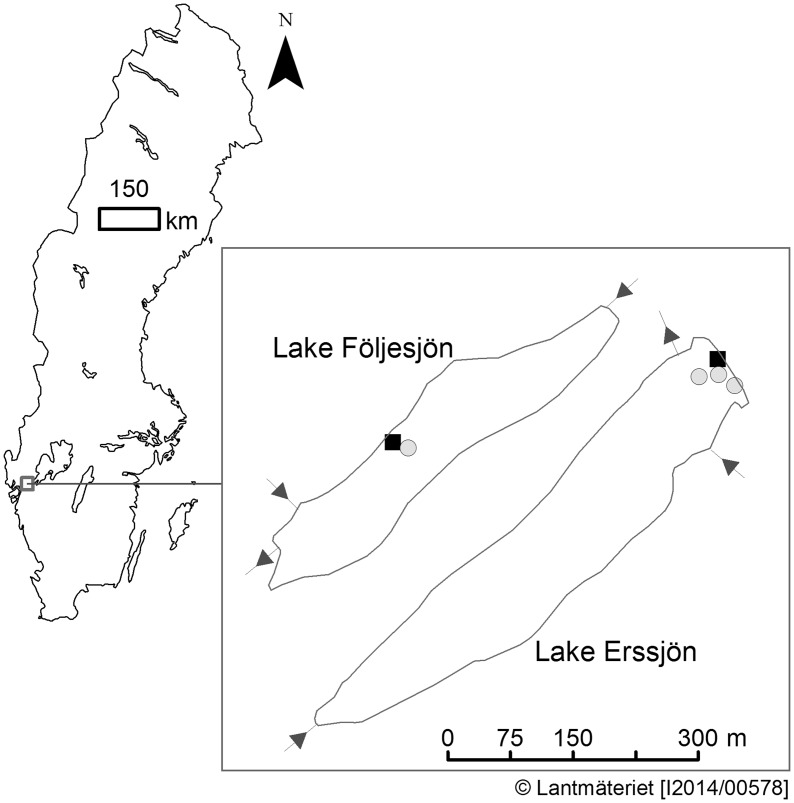
Map of the study area and the sampling sites. Squares represent *Carex rostrata*; circles, *Phragmites australis* (in Erssjön, where the sampling point was moved, the initial one is the left of the two circles).

Five sampling sites (three in Erssjön and two in Följesjön) were chosen considering both accessibility and the abundance of the target plant species (Fig. 1). Within each lake, the sites were all located close to each other to minimize the difference between the sites in other aspects than the dominating plant species. The water depth in each sampling site was ∼10–20 cm. Each site was dominated by one species; *P. australis* or *C. rostrata*. The sampling sites were located in the northern part of Erssjön and in the central part of Följesjön. Initially there were four sampling sites, three of them possible to reach from boardwalks and the fourth site directly reachable from the shore of Erssjön. Due to the high water level in Erssjön in the end of July, the original shore sampling site could not be reached without disturbing the sediment (mixing the sediment can affect the release of CH_4_). This sampling site was therefore moved permanently to a similar nearby area (the fifth site), easily reached from a boardwalk, for the remaining four measurement occasions (Fig. 1).

Measurements in Erssjön were conducted once each hour during 24 h on the two first measurement occasions (9–10 June and 29–30 June 2014) and once every second hour during 24 h on five later occasions (20–21 July, 8–9 August, 29–30 August, 21–22 September and 12–13 October). Samplings in Följesjön took place each hour during 24 h on 1–2 July and every second hour during 24 h on five later measurement occasions (21–22 July, 9–10 August, 30–31 August, 23–24 September and 13–14 October).

### Sampling method and measurement duration

**Figure 2.**
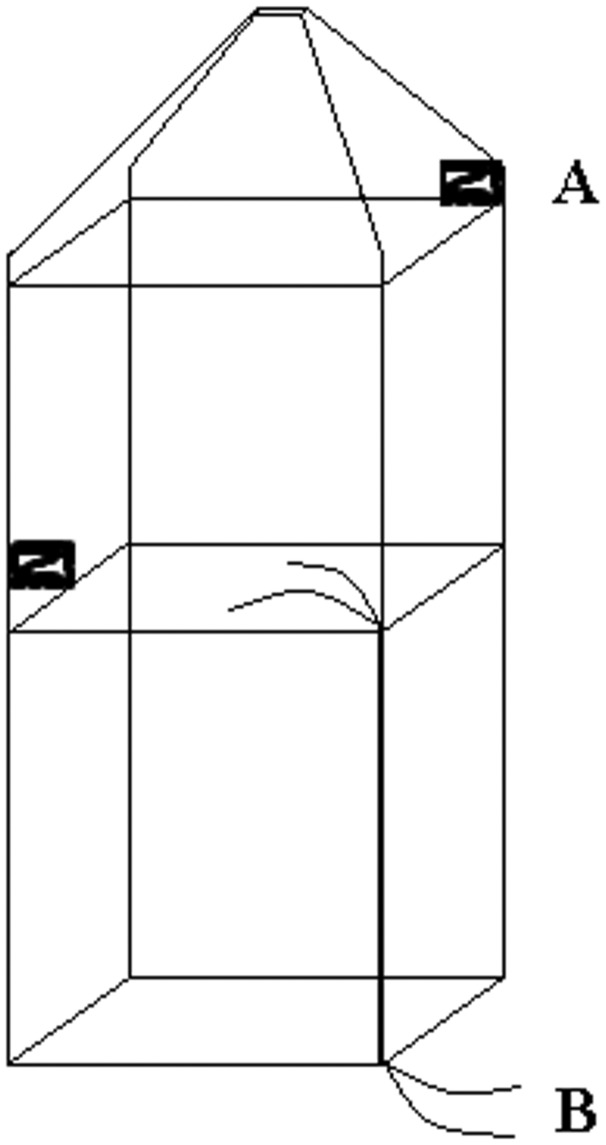
Model of the chamber used for measuring plant-mediated CH_4_ emissions. A is a fan run with a 12 V battery (placed at 100 as well as 60 cm height), B is two transparent PVC tubes transporting the air between the chamber and the LGR (Los Gatos Research (LGR) Ultraportable Greenhouse Gas Analyser, UGGA). The chamber is covered with airtight plastic.

A closed-chamber method was used to measure CH_4_ flux from the areas with emergent aquatic macrophytes. This means that we measured the flux through macrophytes combined with flux through water (the latter being approximately one order of magnitude smaller than the former in the present study). The chamber (d 43 cm, w 43 cm, h 137.5 cm: volume 197.4 L) was made of plastic pipes, built in a rectanglar block shape (Fig. 2) and covered with transparent airtight plastic. The plastic material was made of four layers: 40 pm nylon, 6.5 pm methylene-vinyl-alcohol, 40 pm nylon and 100 pm polyethylene (Otto Nielsen Emballage, 2800 Lyngby, Denmark). The chamber was then put over a stand of emergent aquatic macrophytes in order to collect the gas, floating on Styrofoam rods along the lower edges of the plastic pipe frame and with the plastic entering 5 cm into the water ensuring a good seal. The chamber was equipped with two computer CPU fans, run with 12 V batteries for air circulation and a thermometer to measure the temperature inside the chamber. Gas was sampled from the inside of the chamber through two transparent PVC tubes (outer diameter 5 mm and inner diameter 3 mm) being ∼5 m long. One end was attached inside the chamber and the other end to an LGR-UGGA (Los Gatos Research Ultraportable Greenhouse Gas Analyser), one tube for air entering the LGR-UGGA and the other for outgoing air back to the chamber creating a closed loop between the chamber and the LGR-UGGA. Concentrations of CH_4_ and CO_2_ were logged with the LGR-UGGA every 20 s during 5 min on each plant site. Between measurements, the chamber was removed from the site and vented until the gas levels decreased to the same level as the surrounding air.

### Environmental variables

Air temperature (°C), relative humidity (%) and air pressure (mb) were measured outside the chamber, with a pocket weather station (Anemometer Silva ADC) during each CH_4_ flux measurement. Wind speed (m s^−1^) was obtained from a weather station near each lake (measured wind speed at 30 min intervals at 4.7 m height in Erssjön and at 2.3 m height in Följesjön (data provided by Eric Sahlée and Anna Rutgersson, Department of Earth Sciences, Uppsala University and Leif Klemedtsson, Department of Earth Sciences, University of Gothenburg). Missing values for wind speed were replaced by values from another nearby weather station located on a mire 500 m east of Erssjön (measured at 2.25 m; provided by University of Gothenburg). Light (incoming solar radiation) was measured next to the chamber, with a PAR sensor (HOBO Photosynthetic Active Light Smart Sensor) connected to a HOBO© H21-002 Micro Station Data Logger, which logged incoming light at 10 s intervals. The O_2_ concentration in the water was measured every 15 min at a depth of 0.5 m below the water surface with a HOBO oxygen logger probe (HOBO U26 Dissolved Oxygen Logger). Samples for total phosphorous P, total N, and total Fe and total Mn were collected in 0.5 L polyethylene bottles at 0.5 m depth (one sample per location collected every other week as a part of the regular monitoring program in the area). Total N and P were analysed spectrophotometrically after chemical reduction to ammonia (N) or oxidation to phosphate (P), respectively, and addition of reagent compounds, according to analytical standard ISO 15681-1:2003 and SS-EN ISO 11905-1:1997, respectively. Fe and Mn were analysed using a Nexion 300D ICP-MS (Perkin Elmer).

### Biomass estimation

The aboveground biomass in each chamber was estimated after every sampling day, in total seven times per site. For *P. australis*, plant height was recorded and the number of shoots counted. Biomass in each chamber was estimated from regression equations that were developed by measuring the height of plants and counting shoots in nearby plots, followed by harvesting shoots at the water surface to determine dry weight biomass. For *Carex rostrata*, the shoot density and average height was measured in the chambers on each measurement day. Then, after the measurements, a nearby area (i.e. outside the chambers) with similar shoot density and average height were harvested for biomass determination. The harvested shoots for both species were dried for 48 h at 60 °C and plant biomass dry weight (g DW m^−2^) was determined.

### Calculations

The plant CH_4_ flux was calculated from the linear change in the chambers gas concentration over time according to Bäckstrand *et al.* (2008). The method was modified to fit a larger chamber and a different type of gas measurement. The first minute of each 5 min measurement period was omitted, due to heterogeneous mixing-effects before the gas in the tubing and the LGR measurement cell reflected average chamber gas content. Multiple slopes of the gas concentration versus time were calculated for 2-min periods, each period offset by 20 s. In total seven such 2-min slopes were calculated for each 5-min period. From the seven slopes, the one with the highest *r*^2^-value was chosen for flux estimation.

The following equation was used to calculate the regression slope for each 2-min period, in parts per million per day,

(1) $$\frac{ppm}{day}=\frac{\sum (t_{i}-\overset{-}{t})({ppm}_{i}-\bar{ppm})}{{\sum (t_{i}-\overset{-}{t})}^{2}}$$

where ppm_i_ and *t*_i_ are the starting concentration (in ppm) and starting time for each 2-min period and $\overset{-}{ppm}$ and $\overset{-}{t}$ are the mean of ppm and time for each 2-min period.

The CH_4_ flux (*F*) for the selected 2-min period was calculated according to

(2) $$F=\frac{\frac{ppm}{day}\;\frac{P_{tot}\;}{10^{6}}\;\frac{V}{RT}\;}{A}1000$$

where *ppm/day* is the selected regression slope (Equation 1) from CH_4_ concentration measurements, *P_tot_* is the measured air pressure (atm), *V* is chamber volume (L), *R* is the universal gas constant (0.082056 L atm K^−1^ mol^−1^), *T* is temperature (K) and *A* is the chamber’s base area (m^2^). The value 1000 is used for unit conversion from mol m^−2^ d^−1^ to mmol m^−2^ d^−1^. CH_4_ emissions from the emergent aquatic macrophytes were also calculated per g DW biomass in each measurement site (mmol g DW^−1^ d^−1^). When calculating net flux in this manner, all three types of CH_4_ release from the area covered by the chamber are included, i.e. emission via plants, diffusion across the water surface and minor events of ebullition (larger events were excluded, see below).

### Ebullition

Ebullition releases can be suspected when there is a rapid sudden increase in CH_4_ concentration (also resulting in a reduced R^2^ over the whole 2-min period). Large ebullition events are therefore easily detected in the regression plot of CH_4_ against time. Such an ebullition event occurred one time only, during the last measurement in Följesjön, and this value was excluded. Ebullition events being too small to detect in this way can be considered insignificant compared with the plant-mediated fluxes.

### Diffusive CH_4_ flux

CH_4_ concentration in the water was measured twice in each lake, once in July and once in October, to estimate the diffusive CH_4_ flux. Water samples were taken with a 10 mL plastic syringe and ∼5 mL water was collected to rinse the syringe prior to sampling. During this rinsing, care was taken to also remove air bubbles from the syringe. When the rinsing water had been discarded, 5 mL water were sampled 3 cm below the surface, and, after visually confirming absence of bubbles, the sample was injected into a pre-capped vial (20 mL) filled with 100 µL H_3_PO_4_ and N_2_ at atmospheric pressure. CH_4_ concentration in the water was analysed in the laboratory by a gas chromatograph (Agilent 6890 with Haysep N column and flame ionization detector calibrated with certified gas standards).

The diffusive CH_4_ flux can be expressed with the equation:

(3) $$F=k\times \left(C_{w}-C_{eq}\right)$$

where *F* is diffusive CH_4_ flux in mmol m^−2^ d^−1^, *k* is the gas transfer velocity (m d^−1^), *C_w_* is the measured CH_4_ concentration in the water (mmol m^−3^), and *C_eq_* is the CH_4_ concentration in the water if it was in equilibrium with the air concentration (estimated from air concentrations and Henry’s Law). The *k*-value was derived from independent flux and concentration measurements on the nearby open water (see, e.g. Bastviken *et al.* 2010 and Natchimuthu *et al.* 2016 for method details). This can be considered to yield overestimated diffusive flux as previous work has indicated that *k*-values are much lower in stands of macrophytes where the water surface is wind-sheltered than for open water (Kosten *et al.* 2016; Attermeyer *et al.* 2016).

### Statistical analyses

Methane emissions were analysed in three ways, with focus (i) on the possible existence of diel patterns; (ii) simple relationships between mean diel CH_4_ flux per m^2^ and different environmental variables; and (iii) on modelling all data using all explanatory variables.

To evaluate whether there were consistent diel patterns in CH_4_ emissions among the 25 daily time series (one incomplete series excluded), we used intra-class correlation (Rusak *et al.* 1999). We first divided the day into 10 time classes, calculating an average per class (there were 1–4 values per time class). These calculations were conducted with SPSS 24, using the two-way mixed model, with absolute agreement.

The relationships between mean diel CH_4_ flux and temperature, wind, light and net CO_2_ exchange, respectively, were evaluated using linear regression. Relationships for which the slopes had *P*-values < 0.05 were considered significant.

In approach (iii), all 5 min flux measurements (*N*  =  288) were analysed using a generalized linear model (GLZ, McCullagh and Nelder 1989) with a log-link function and Gamma distribution using the software R (R Core Team 2013). Explanatory variables were lake, plant species, air temperature, temperature inside the chamber, light, air pressure, air humidity, wind speed, plant biomass, CO_2_ flux, date and time, where date and time were treated as separate variables in the model. A quadratic term of time and date was included, as we expected a nonlinear response of CH_4_ flux during the day and over the growing season (based on previous studies, a peak in plant CH_4_ emission was expected during the day and highest emissions were expected in the summer during the peak of the growing season; Van der Nat *et al.* 1998; Kankaala *et al.* 2005). An AIC-based model averaging and selection approach was used (Symonds and Moussalli 2011).

In model selection and averaging, candidate models are the set of all possible models that are nested within the full model and the AIC values (Akaike Information Criterion; Akaike 1974) for candidate models are used as a measure of model fit. The difference in AIC between models is used to rank models, to determine the importance of explanatory variables and to estimate regression coefficients. Model selection was implemented using the MuMIn-package (Barton 2016), and continuous explanatory variables were standardized to have mean = 0 and SD = 0.5 prior to analysis. Correlations between explanatory variables were calculated to assess the degree of collinearity. Humidity and temperature inside the chamber were excluded from the model due to their high correlation with air temperature (*r* > 0.7). The fitted full model was tested for collinearity (Zuur *et al.* 2010) with the vif function (car-package; Fox and Weisberg 2011); detecting only minimal collinearity (vif < 3). Furthermore, residuals from the fitted model did not show heteroscedasticity.

Linear regression is sensitive to un-equal sampling intensity along the explanatory gradients. The greater number of measurements in the beginning of the growing season might bias the results and thus lead to erroneous conclusions. Therefore, every second value, from a total of 24 measurements in 9–10 June, 29–30 June and 1–2 July were omitted, in order to have a consistent dataset with twelve measurements per day. Autocorrelation between consecutive measurement during a day was detected with the ar-function (in the stats-package in R), for days with 24 measurements. However, there was less autocorrelation when every second value was deleted, so no further process for handling autocorrelation in the model was needed. 

## Results

### Plant mediated and diffusive CH_4_ emissions

Our flux estimates ranged from 1 to 87 mmol m^−2^ d^−1^ and 1 to 73 mmol m^−2^ d^−1^ for *P. australis* and *C. rostrata*, respectively (Table 2; equivalent to 0.7–58 and 0.7–49 mg CH_4_ m^−2^ h^−1^). When calculating CH_4_ emissions from the emergent aquatic macrophytes per m^2^, the diffusive flux of CH_4_ and ebullition were included in the total emission. The measured diffusive emissions ranged from 0.11 to 2.46 mmol m^−2^ d^−1^, which represented 1–22 % of the total mean emission from the emergent aquatic macrophytes on the day that measurements were made (Table 2). In an extensive, previous 2-year study of the two lakes, median diffusive fluxes ranged from 0.1 to 1.0 mmol m^−2^ d^−1^ (Natchimuthu *et al.* 2016) confirming that diffusive flux, where plants were present, was small relative to plant-mediated fluxes. Further, these numbers are most likely overestimates of the diffusive flux at the measurement plots because gas exchange rates are lower among plants than for open water (see Methods section).

**Table 2:** Maximum, minimum and mean of CH_4_ emission (mmol m^−2^ d^−1^) for *P. australis* and *C. rostrata* in Lake Erssjön and Lake Följesjön, throughout the study period in 2014 (*n* = 7).

CH_4_ emission (mmol m^−2^ d^−1^)			Measurement occasion						
			9–10 June	29 June to 2 July	20–22 July	8–10 Aug.	29–31 Aug.	21–24 Sep.	12–14 Oct.
Erssjön	*P. australis*	Maximum	86.7	15.4	37.6	38.0	26.8	11.7	5.46
		Minimum	6.60	2.39	1.87	6.07	6.78	0.94	1.60
		Mean	21.6	4.78	11.1	15.4	12.7	4.11	3.42
	*C. rostrata*	Maximum	20.7	10.8	–	17.8	14.5	4.21	5.10
		Minimum	7.20	5.20	–	9.09	8.2	2.01	1.05
		Mean	14.7	6.75	–	13.0	10.0	2.88	2.14
Följesjön	*P. australis*	Maximum	–	44.1	55.4	77.6	40.1	59.2	35.40
		Minimum	–	2.69	19.3	11.7	8.57	1.98	5.74
		Mean	–	16.9	32.6	49.9	19.8	15.6	13.7
	*C. rostrata*	Maximum	–	31.5	73.0	54.1	29.1	28.4	34.2
		Minimum	–	9.90	24.6	20.6	8.29	4.12	4.05
		Mean	–	18.6	37.0	32.5	16.0	10.6	15.1

### Variability between lakes, plant species and measurement days

**Figure 3.**
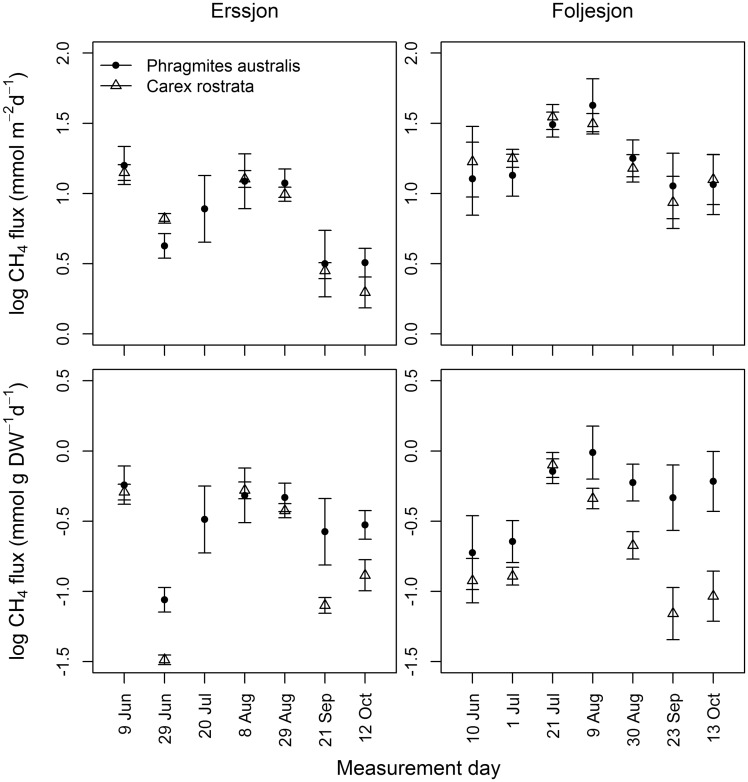
Fluxes of CH_4_ (diel average with CI_95%_, *n*** **=** **7) from sites dominated by *Phragmites australis* and *Carex rostrata* in Erssjön and Följesjön during June to October 2014. Top graphs show fluxes calculated per m^2^ and bottom graphs fluxes calculated per g DW.

The 24-h mean CH_4_ emissions per m^2^ differed between lakes with higher fluxes in Följesjön (Fig. 3). There were no differences in these CH_4_ emissions (per m^2^) between *P. australis* and *C. rostrata* within the same lake (Fig. 3). The highest 24-h mean fluxes occurred in the summer (June–August) and the lowest fluxes were recorded during September and October in both lakes (Table 2 and Fig. 3). There was a 5-fold difference between measurement days with highest and lowest flux for both plants species. The variability between measurement days were consistent for both *P. australis* and *C. rostrata*, i.e. low and high fluxes occurred during the same days for both species.

When expressing 24-h mean fluxes per g DW (instead of per m^2^) the temporal pattern among measurement days remains, but there were differences between plots with different plants, with lower fluxes per g DW from *Carex rostrata* stands some of the days (Fig. 3).

### Diel variation in CH_4_ emission

**Figure 4.**
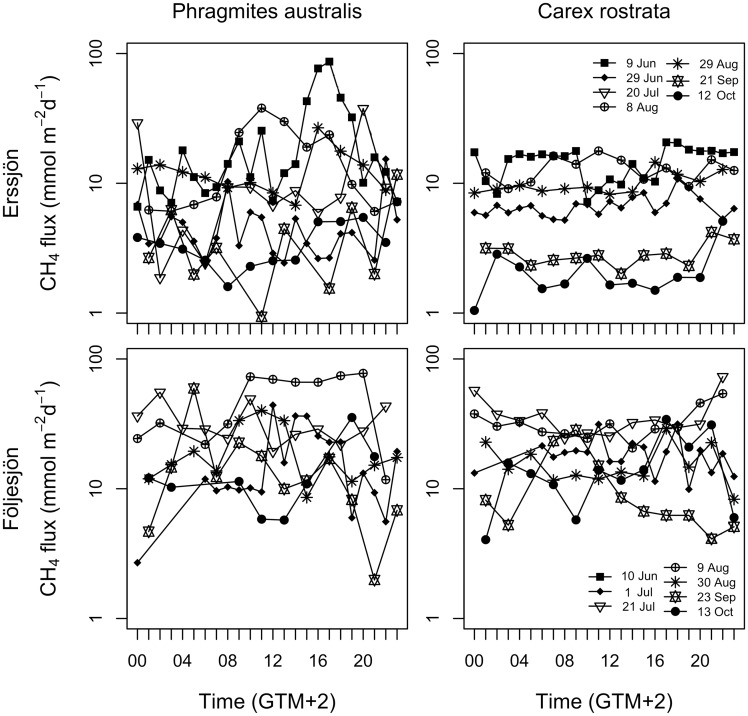
Diel variation in CH_4_ fluxes from sites dominated by *Phragmites australis* and *Carex rostrata* in Erssjön and Följesjön, for each measurement occasion during the growing season (June to October, *n*** **=** **7). Measured in Swedish summertime (GMT** **+** **2).

There was variability in CH_4_ emissions within days (Fig. 4). However, recurrent peaks in CH_4_ emissions at the same time on the different measurement days (i.e. a consistent diel pattern) were not found for the two species (intra-class correlation coefficient based on both species: 0.055, *P* = 0.316; coefficients for *P. australis* and *C. rostrata* were 0.014 and 0.017, respectively). Variation during the days was higher for *P. australis* than for *C. rostrata* in Erssjön, where *P. australis* had both higher and lower emissions registered each measurement day compared to *C. rostrata* (Table 2).

### Mean daily CH_4_ flux and single environmental variables

**Figure 5.**
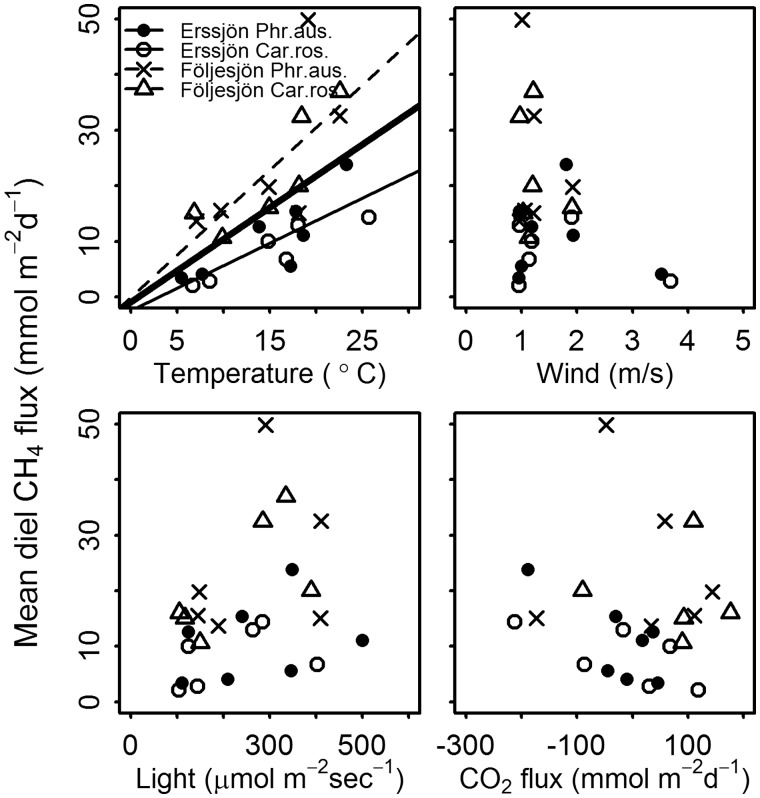
Mean diel CH_4_ emissions in relation to air temperature (*t*_air_), wind, light and CO_2_ flux (i.e. net ecosystem CO_2_ exchange) for measurement plots with *Phragmites australis* and *Carex rostrata* in Lake Erssjön and Lake Följesjön, respectively. Lines show significant linear regressions. The CH_4_ flux (F_CH4_; mmol m^−2^ d^−1^) were similar among species (see text) and the dashed and thin solid lines represent the regressions for both species combined in Följesjön (F_CH4_ = 1.529·*t*_air_ – 0.068; *P* = 0.009; adj. *R*^2^ = 0.46) and Ersjön (F_CH4_ = 0.811·*t*_air_ – 2.487; *P* < 0.001; adj. *R*^2^ = 0.63), respectively. The thick solid line denotes the overall regression for all data (F_CH4_ = 1.136·*t*_air_ – 0.984; *P* = 0.003; adj. *R*^2^ = 0.30). No significant relationships were found for wind, light or CO_2_ flux.

There was a significant relationship between mean daily CH_4_ emissions per m^2^ and mean diel temperature (Fig. 5). There were no significant relationships between diel means of wind speed, light levels or net CO_2_ exchange and CH_4_ emissions (Fig. 5).

### Modelling CH_4_ flux

The results from the regressions of daily means on CH_4_ emission per m^2^ were confirmed by the AIC-based GLZ model selection and averaging procedure using *N* = 288 data points. The analysis found that models that contained lakes and air temperature were better at explaining methane flux (Table 3). These variables were included in the all best fitting candidate models (i.e. relative importance = 1). The model average parameter estimate indicated that methane flux increased with temperature and was higher in Följesjön than in Erssjön (Table 3). An increase in light and wind had a (significant) negative effect on CH_4_ emissions, while plant species, biomass dry weight, NEE of CO_2_, and time of day were much less important (Table 3).

**Table 3:** The effect of categorical and continuous variables on emergent aquatic macrophytes CH_4_ emissions per m^2^. Data from the full generalized linear model (GLZ; estimate) and from a model selection and averaging of nested candidate GLZ-models with standardized continuous variables (model average estimate, standard error (SE), *z*-value, 95 % confidence interval (CI) and relative importance for variables). Values marked with bold text are statistically significant.

Methane flux (mmol m^−2^ d^−1^)	Estimate	Standardized values					
		MAE*	SE	*z*-value	CI interval		RI*
					Lower	Upper	
Intercept	2.502e + 07	2.637	0.069	37.962	**2.501**	**2.773**	
Lake Erssjön (compared with Lake Följesjön)	−9.995e − 01	−0.991	0.097	10.202	**−1.181**	**−0.800**	**1.0**
Air temperature (C°)	5.826e − 02	0.896	0.172	5.182	**0.557**	**1.235**	**1.0**
Light (µmol m^−2^ s^−1^)	−2.929e − 04	−0.242	0.111	2.178	−**0.459**	−**0.024**	**0.9**
Wind (m/s)	−8.890e − 02	−0.218	0.095	2.291	−**0.404**	−**0.031**	**0.93**
Date 1 (Measurement day)	1.894e − 05	−0.279	0.135	2.057	−**0.545**	−**0.013**	**0.9**
Date 2 (Measurement day)**	−6.740e − 15	−0.342	0.187	1.821	−0.710	0.026	0.9
*P. australis* (compared to *C. rostrata*)	1.488e − 01	0.172	0.090	1.913	−0.004	0.348	0.77
Air pressure (atm)	6.357e − 01	0.028	0.102	0.270	−0.172	0.227	0.27
Biomass (g DW)	−1.011e − 03	−0.145	0.115	1.259	−0.370	0.081	0.50
NEE (net ecosystem exchange of CO_2_)	9.841e − 05	0.092	0.085	1.077	−0.075	0.259	0.48
Time 1 (Time of the day)	−3.516e − 02	−0.013	0.089	0.148	−0.189	0.163	0.12
Time 2 (Time of the day)**	1.234e − 11	0.115	0.238	0.481	−0.353	0.582	0.12

* MAE (Model Average Estimate) and RI (Relative Importance).

** Quadratic term.

## Discussion

### No clear diel flux patterns

While our flux values correspond with previously recorded ranges for CH_4_ flux via *P. australis* and various *Carex* species (mean fluxes reported to 0–80 and 0–33 mg CH_4_ m^−2^ h^−1^; examples of ours and previously reported fluxes provided in Table 4), our results challenge common views on diel variability. We found that peaks in plant-mediated CH_4_ emissions occurred during all hours of the day but were not very strong, nor did they dominate the total daily flux (Fig. 4). Furthermore, the diel patterns for *P. australis* did not coincide with patterns for *C. rostrata*.

**Table 4:** Examples of studies addressing diel variability in CH_4_ flux from areas with *Phragmites australis* or *Carex* species.

Type of envrionment and location	Dominating plants	Time period	Mean flux range (mg CH_4_ m^−2^ d^−1^)		Diel flux pattern	No of diel cycles reported	Method used for flux measurements	Source
			Low	High				
Lakeshore sites, central Finland	*P. australis*	July to October	0	32	24h mean = 0.58 to 0.91 times the mean for daytime flux. Most shallow sites showed less clear diel variability	many (unclear)	Automatic flux chamber; ca 5 measurements per diel cycle	Juutinen et al. (2004)
Constructed wetland, The Netherlands	*P. australis*	Unclear	20	80	Flux positively related with PAR. Up to 2-fold difference between day and night.	1 (unclear)	Chambers connceted to analyzer. 15-50 min enclosure.	Van der Nat et al. (1998)
Eutrophic lake shore, sourthern Finland	*P. australis*	May to September	1	18	Highest flux at noon in August and September. Irregular patterns other times.	7	manual sampling over 9 min every 6th h.	Käki et al. (2001)
Temperate fen, Germany	*P. australis*	9 days, August	3.5	11	incrasing mean fluxes from 06-14 (2-fold increase)	1-6 (unclear)	Transparent chambers; 40 min incubation; syringe sampling. Time period of 06-14 studied.	Günther et al. (2014)
Two fens in Belarus; focus on shallow water plots	*P. australis*	2 days, June, August	2	20	Fluxes in both transparent and dark chambers increased with PAR. >2-fold higher flux during daytime.	2	Chamber; discrete samples taken over 8-12 min	Minke et al. (2014)
March, Nebraska, USA	*P. australis*	65 days, July–September	8	25	Fluxes highest midmorning-noon. 2-fold higher flux during daytime.	4; 2-day periods	Eddy covariance	Kim et al. (1998)
Hemiboreal lake-wetland, southwest Sweden	*P. australis*	June to October	11	33	No consistent diel pattern	6 x 2	Chambers connected to analyzer. 5 min enclosure; fluxes measured every 2h.	This study
Hemiboreal lake shore, southwest Sweden	*P. australis*	June to October	3	58	No consistent diel pattern	7 x 2	Chambers connected to analyzer. 5 min enclosure; fluxes measured every 2h.	This study
Lakeshore sites, central Finland	*Carex* spp.	August	0	32	No strong diel pattern	12	Automatic flux chamber; ca 5 measurements per diel cycle	Juutinen et al. (2004)
Temperate fen, Germany	*C. acutiformis*	9 days, August	8	11	No clear diel patterns	1–6 (unclear)	Transparent chambers; 40 min incubation; syringe sampling; time period of 06-14 studied.	Günther et al. (2014)
Eutrophic marsh, China	*C. lasiocarpa*	August	20	33	Increasing flux from 03 to 09 (up to 1.5-fold increase)	3	Flux chambers sampled manually; 30 min enclosure time; 3 h enclosure frequency	Ding et al. (2004)
Hemiboreal lake-wetland, southwest Sweden	*C. rostrata*	June to October	3	16	No consistent diel pattern	6	Chambers connceted to analyzer. 5 min enclosure; fluxes measured every 2 h.	This study
Hemiboreal lake shore, southwest Sweden	*C. rostrata*	June to October	3	14	No consistent diel pattern	6	Chambers connceted to analyzer. 5 min enclosure; fluxes measured every 2 h.	This study

Previous studies of diel variability often found clear flux peaks and it has been suggested that predominating convective flux mechanisms actively regulated by the plants lead to a stronger diel cycle than when diffuse and more passive flux through the plant dominates (Whiting and Chanton 1996; Juutinen *et al.* 2004; Duan *et al.* 2005). The timing of the observed flux peaks has been shown to vary between species (Brix *et al.* 1996; Whiting and Chanton 1996). However, it is sometimes unclear how stable the diel patterns were over time when patterns from only a single day are reported (e.g. Whiting and Chanton 1996; Van der Nat *et al.* 1998; Ding *et al.* 2004). The present study reports diel measurements during seven measurement days, and clearly shows that the diel patterns vary considerable among them.

Gas exchange by plants is thought to be controlled by stomatal opening allowing gas diffusion into or out from the aerenchyma, in some species facilitated by pressure-driven convection. The convection is controlled by internal/external differences in relative humidity (RH) or temperature, or by wind (reviewed by Sorrell and Brix 2013 and Armstrong and Armstrong 2014). Aquatic plants do not need to save water and can keep stomata open during night (Käki *et al.* 2001). Accordingly, diffusion-driven gas exchange is often found to lack clear diel patterns for emergent aquatic plants. The absence of strong and consistent diel patterns was therefore expected for *C. rostrata*, but was surprising for *P. australis*, which commonly is found to have gas exchange that is positively related to insolation (Table 4). Light has been suggested to influence convection-driven diel variability in fluxes, presumably by affecting RH and temperature gradients. The detailed AIC-based GLZ model selection and averaging showed that light and wind were of intermediate importance and had a weak negative correlation with CH_4_ flux (Table 3). The GLZ includes all time points and combines both diel and seasonal data so the wind and light correlations may not primarily be associated with diel variability. However, it is possible that the long days and short (if any) dark periods during summertime at northern latitudes may make the diel gas exchange patterns less predictable than at more southern latitudes having more pronounced day-night differences in light. For example, diel patterns related to the light cycle were observed from measurements in August and September only (when nights get darker) at a lakeshore site in Finland, while patterns were unclear during May to July (brighter nights) (Käki *et al.* 2001). Altogether, our result indicated that CH_4_ fluxes from *P. australis* may not always show distinct diel patterns, and that further work addressing diel variability at different locations and under different conditions is needed.

### What controls plant-mediated CH_4_ emissions?

Methane emissions from both species were on average three times higher in Följesjön than in Erssjön (Table 1). This is in line with the study by Natchimuthu *et al.* (2016) that concludes that the amount of released CH_4_ from the open water areas in Följesjön exceeded open water fluxes from Erssjön. Possible explanations include that Följesjön was very shallow, had thick organic-rich sediments, and a higher standing crop of emergent vegetation leading to a high production of organic matter substrates for methanogenesis per unit area. This is a speculation based on the overall abundance of macrophytes and bulk organic matter, as measuring the in-situ production of the specific original plant material fuelling microbial communities including methanogens in sediments is difficult and beyond the scope of this study. However, variables associated with high primary production or high levels of organic matter are known to stimulate CH_4_ production and fluxes (e.g. Segers 1998; Bastviken *et al.* 2004; Bridgham *et al.* 2013), giving some support for this potential explanation of the higher overall fluxes from Följesjön.

The temporal variability of CH_4_ emissions within each system was related to temperature (Table 3 and Fig. 5). Although we used air temperature, the temperature of the sediment was probably more important (Johansson *et al.* 2004; Duan *et al.* 2005), which provides an additional reason for the lake differences; Följesjön was the shallowest lake, hence likely to have a higher sediment temperature during the growing season. The sediment in a shallower lake with a smaller water volume is also likely to respond faster to changes in air temperatures. Several studies have demonstrated a relationship between CH_4_ emissions and sediment temperature, in accordance with the high temperature-sensitivity of methanogenesis (Segers 1998; Wang and Han 2005; Duc *et al.* 2010; Yvon-Durocher *et al.* 2014; Turetsky *et al.* 2014). In contrast, oxidation of CH_4_ seems unaffected by temperature (most often limited by substrate supply; Nykänen *et al.* 1998; Duc *et al.* 2010).

It should be noted that temperature can have a direct influence on process rates, as discussed above, but also is correlated with seasonal fluctuations and production of organic substrates for methanogenesis (Laanbroek 2010; Carmichael *et al.* 2014). Several studies reported maximum biomass during the growing season as the key explanatory factor for CH_4_ emissions (Hirota *et al.* 2004; Juutinen *et al.* 2004; Kankaala & Bergström 2004). However, in the present study and in that by Kankaala *et al.* (2004), biomass could not explain the temporal variability in CH_4_ emissions per m^2^ (Table 3), suggesting that direct temperature effects or organic matter from other sources (e.g. the catchment) could also influence fluxes.

Wind speed and light were shown to weakly affect CH_4_ emissions from *P. australis* and *C. rostrata* negatively in both lakes (Table 3). In contrast, many previous studies showed that light had a positive effect on the CH_4_ emission from *P. australis* in relation to diel patterns (discussed above; Table 4). According to Duan *et al.* (2005) light can affect CH_4_ emissions also on a larger scale (e.g. seasonal patterns) during the period when the emergent aquatic macrophytes transport gas with convective flow (light-dependent plant activity suggested to regulate gas exchange). In the study by Juutinen *et al.* (2004), light could explain 39–73 % of the variation in plant-mediated CH_4_ emissions during the middle of the growing season. However, light often co-varies with temperature, other weather variables, and primary production or net CO_2_ exchange, and it is often unclear if effects from light, temperature and other variables can be separated. Our AIC-based GLZ model selection and averaging procedure is one way to approach this challenge and it clearly showed that temperature was more strongly related to the flux than light and wind, with no clear link between CH_4_ flux and net CO_2_ exchange.

### Did plant species identity influence emissions?

Plant species are expected to differ in the amount of CH_4_ they can emit due to, e.g. the extent of their root system, the amount of biomass they have, and differences in flux modes (diffusive or convective). Plant-mediated CH_4_ emissions are usually reported per m^2^, a unit useful for scaling up estimates of emission. On the other hand, CH_4_ emissions per plant biomass unit may be more appropriate if focusing on emission mechanisms and plant architecture. In the present study we therefore reported both. We expected that the convective flux mode (*P. australis*) should cause higher emissions than the passive one (*C. rostrata*). However, sites with *P. australis* and *C. rostrata* always showed similar fluxes per m^2^ in the same systems on nearby sites (Fig. 3). Further, if comparing results from the lakes in Fig. 3, it is clear that the same flux rates could be sustained by widely different plant densities (maximum biomass values for *P. australis* were 25 and 60 g m^−2^ and for *C. rostrata* 204 and 139 g m^−2^ for Ersjön and Följesjön, respectively). The differences found in the fluxes per g DW plant biomass therefore largely reflected differences in standing crop biomass of the two species, and did not provide any clear information about fluxes per se or about flux regulation. Hence, most of the variability in plant-mediated flux per m^2^ appeared related to temperature and between-lake differences in environmental conditions as discussed above, while plant species/flux mode had, at most, a minor influence. We suggest additional studies in multiple systems to investigate under what conditions plant species communities affect fluxes, while trying to separate effects of plant species and other variables regulating CH_4_ production on the specific site.

## Conclusions

In summary, and in contrast to many previous studies on CH_4_ emissions via aquatic macrophytes, we found the following:

1. Diel variability in CH_4_ fluxes from *P. australis* and *C. rostrata*, representing plants with convective and diffusive flux modes, respectively, were irregular in magnitude and timing and unpredictable based on data from seven days distributed from June to October.
2. The 24-h mean fluxes per m^2^ on nearby sites were similar between species and highly temperature-dependent. Differences between lakes were consistent with factors influencing system CH_4_ fluxes such as macrophyte standing biomass and water depth. No clear influence of other studied potential predictors such as light, wind, pressure, and NEE of CO_2_ were found.
3. All available data from this study indicate similar total CH_4_ flux per m^2^ from plots with *P. australis* and *C. rostrata*. Hence, fluxes were not controlled by the dominating plant species but more likely by the overall CH_4_ production in the systems.

Given these results, the presence and importance of diel variability in plant fluxes, as well as the importance of macrophyte species composition under various conditions needs to be re-evaluated and assessed systematically over time across a range of environments and species.

## Sources of Funding

Grants from the Swedish Research Council and the European Research Council to DB (VR 2012-48, VR 2011-3575, ERC no. 725546), and from Stiftelsen Oscar och Lili Lamms Minne to LT (EX2014-0010) supported this study. The Skogaryd Research Catchment is part of the SITES infrastructure, which received funding from two Swedish research council: VR and Formas.

## Contributions by the Authors

D.B. and P.M. conceived the study; all authors contributed to the design; L.T. collected the data; L.T. and L.W. analysed the data; all authors contributed to evaluation and interpretation of the results; L.T. drafted the text (as part of a MSc thesis) with all authors contributing substantially to the current version.

## Conflict of Interest Statement

 None declared.

## References

[plx029-B1] Akaike H. 1974 A new look at the statistical model identification. IEEE Transactions on Automatic Control 19:716–723.

[plx029-B2] Armstrong J, Armstrong W. 1991 A convective through-flow of gases in *Phragmites australis* (Cav.) Trin. Ex Steud. Aquatic Botany 39:75–88.

[plx029-B3] Armstrong W, Armstrong J. 2014 Plant internal oxygen transport (diffusion and convection) and measuring and modelling oxygen gradients In: van Dongen JT, Licausi F, eds. Low-oxygen stress in plants. Plant Cell Monographs, Vol. 21 Vienna: Springer, 267–298.

[plx029-B4] Attermeyer K, Flury S, Jayakumar R, Fiener P, Steger K, Arya V, Wilken F, van Geldern R, Premke K. 2016 Invasive floating macrophytes reduce greenhouse gas emissions from a small tropical lake. Scientific Reports 6:20424. 26846590 10.1038/srep20424 PMC4742780

[plx029-B5] Bäckstrand K, Crill PM, Mastepanov M, Christensen TR, Bastviken D. 2008 Non-CH_4_ volatile organic compound flux from a subarctic mire in Northern Sweden. Tellus 60B:226–237.

[plx029-B6] Barton K. 2016 *MuMIn: Multi-Model Inference. R package version 1.15.6* https://CRAN.R-project.org/package=MuMIn.

[plx029-B7] Bastviken D. 2009 CH_4_ In: Likens GE, ed. *Encyclopedia of Inland waters*, Vol. 2, Oxford: Elsevier, 783–805.

[plx029-B8] Bastviken D, Cole J, Pace M, Tranvik L. 2004 CH_4_ emissions from lakes: dependence of lake characteristics, two regional assessments, and global estimate. Global Biogeochemical Cycles 18:GB4009.

[plx029-B9] Bastviken D, Cole JJ, Pace ML, Van de Bogert MC. 2008 Fates of CH_4_ from different lake habitats: connecting whole-lake budgets and CH_4_ emissions. Journal of Geophysical Research 113:G02024.

[plx029-B10] Bastviken D, Santoro AL, Marotta H, Pinho LQ, Calheiros DF, Crill P, Enrich-Prast A. 2010 CH_4_ emissions from Pantanal, South America, during the low water season: toward more comprehensive sampling. Environmental Science and Technology 44:5450–5455. 20568738 10.1021/es1005048

[plx029-B11] Bastviken D, Tranvik LJ, Downing JA, Crill PM, Enrich-Prast A. 2011 Freshwater methane emissions offset the continental carbon sink. Science 331:50. 21212349 10.1126/science.1196808

[plx029-B12] Bergström I, Mäkelä S, Kankaala P, Kortelainen P. 2007 CH_4_ efflux from littoral vegetation stands of southern boreal lakes: an upscaled regional estimate. Atmospheric Environment 41:339–351.

[plx029-B13] Bhullar GS, Iravani M, Edwards PJ, Olde Venterink H. 2013 Methane transport and emissions from soil as affected by water table and vascular plants. BMC Ecology 13:32. 24010540 10.1186/1472-6785-13-32 PMC3847209

[plx029-B14] Bridgham SD, Cadillo-Quiroz H, Keller JK, Zhuang QL. 2013 Methane emissions from wetlands: biogeochemical, microbial, and modeling perspectives from local to global scales. Global Change Biology 19:1325–1346. 23505021 10.1111/gcb.12131

[plx029-B15] Brix H, Sorrell BK, Schierup HH. 1996 Gas fluxes achived by in situ convective flow in *Phragmites australis*. Aquatic Botany 54:151–163.

[plx029-B16] Carmichael MJ, Bernhardt ES, Bräuer SL, Smith WK. 2014 The role of vegetation in CH_4_ flux to the atmosphere: should vegetation be included as a distinct category in the global CH_4_ budget?. Biogeochemistry 119:1–24.

[plx029-B17] Chanton JP, Whiting GJ. 1996 CH_4_ stable isotopic distributions as indicators of gas transport mechanisms in emergent aquatic plants. Aquatic Botany 54:227–236.

[plx029-B18] Ciais P, Sabine C, Bala G, Bopp L, Brovkin V, Canadell J, Chhabra A, DeFries R, Galloway J, Heimann M, Jones C,L, Quéré C, Myneni RB, Piao S, Thornton P. 2013 Carbon and other biogeochemical cycles In: Climate Change 2013: the Physical Science Basis. Contribution of Working Group I to the Fifth Assessment Report of the Intergovernmental Panel on Climate Change. Cambridge, New York, UK, USA: Cambridge University Press.

[plx029-B19] Conrad R. 2009 The global methane cycle: recent advances in understanding the microbial processes involved. Environmental Microbiology Reports 1:285–292. 23765881 10.1111/j.1758-2229.2009.00038.x

[plx029-B20] Ding W, Cai Z, Tsuruta H. 2004 Diel variation in CH_4_ emissions from the stands of *Carex lasiocarpa* and *Deyeuxia angustifolia* in a cool temperate freshwater marsh. Atmospheric Environment 38:181–188.

[plx029-B21] Ding W, Cia Z, Tsuruta H. 2005 Plant species effects on CH_4_ emissions from freshwater marshes. Atmospheric Environment 39:3199–3207.

[plx029-B22] Duan X, Wang X, Mu Y, Ouyang Z. 2005 Seasonal and diurnal variations in CH4 emissions from Wuliangsu Lake in arid regions of China. Atmospheric Environment 39:4479–4487.

[plx029-B23] Duc NT, Crill P, Bastviken D. 2010 Implications of temperature and sediment characteristics on methane formation and oxidation in lake sediments. Biogeochemistry 100:185–196.

[plx029-B24] Fox J, Weisberg S. (2011) An {R} companion to applied regression, 2nd edn Thousand Oaks CA: Sage http://socserv.socsci.mcmaster.ca/jfox/Books/Companion).

[plx029-B25] Günther A, Jurasinski G, Huth V, Glatzel S. 2014 Opaque closed chambers underestimate methane fluxes of *Phragmites australis* (Cav.) Trin. Ex Steud. Environmental Monitoring and Assessment 186:2151–2158. 24213640 10.1007/s10661-013-3524-5

[plx029-B26] Hirota M, Tang Y, Hu Q, Hirata S, Kato T, Mo W, Cao G, Mariko S. 2004 CH_4_ emissions from different vegetation zones in a Qinghai-Tibetan plateau wetland. Soil Biology and Biochemistry 36:737–748.

[plx029-B27] Hyvönen T, Ojala A, Kankaala P, Martikainen P. 1998 CH_4_ release from stands of water horsetail (*Equisetum fluviatile*) in a boreal lake. Freshwater Biology 40:275–284.

[plx029-B28] Joabsson A, Christensen TR, Wallén B. 1999 Vascular plant controls on CH_4_ emissions from northern peatforming wetlands. Trends in Ecology and Evolution 14:385–388. 10481199 10.1016/s0169-5347(99)01649-3

[plx029-B29] Johansson AE, Gustavsson AM, Öqvist MG, Svensson BH. 2004 Methane emissions from constructed wetland treating wastewater: seasonal and spatial distribution and dependence on edaphic factors. Water Research 38:3960–3970. 15380986 10.1016/j.watres.2004.07.008

[plx029-B30] Juutinen S, Alm J, Larmola T, Huttunen JT, Morero M, Saarnio S, Martikainen PJ, Silvola J. 2003 Methane (CH_4_) release from littoral wetlands of boreal lakes during an extended flooding period. Global Change Biology 9:413–424.

[plx029-B31] Juutinen S, Alm J, Larmola T, Saarnio S, Martikainen PJ, Silvola J. 2004 Stand-specific diurnal dynamics of CH_4_ fluxes in boreal lakes: Patterns and controls. Journal of Geophysical Research 109:D19313.

[plx029-B32] Käki T, Ojala A, Kankaala P. 2001 Diel variation in methane emissions from stands of *Phragmites australis* (Cav.) Trin. ex Steud. and *Typha latifolia* L. in a boreal lake. Aquatic Botany 71:259–271.

[plx029-B33] Kankaala P, Bergström I. 2004 Emission and oxidation of CH_4_ in *Equisetum fluviatile* stands growing on organic sediment and sand bottoms. Biogeochemistry 67:21–37.

[plx029-B34] Kankaala P, Ojala A, Käki T. 2004 Temporal and spatial variation in CH_4_ emissions from a flooded transgression shore of a boreal lake. Biogeochemistry 68:297–311.

[plx029-B35] Kankaala P, Käki T, Mäkelä S, Ojala A, Pajunen H, Arvola L. 2005 CH_4_ efflux in relation to plant biomass and sediment characteristics in stands of three common emergent macrophytes in boreal mesoeutrophic lakes. Global Change Biology 11:145–153.

[plx029-B36] Kim J, Verma SB, Billesbach DP, Clement RJ. 1998 Diel variation in CH_4_ emission from midlatitude prairie wetland: significance of convective throughflow in *Phragmites australis*. Journal of Geophysical Research 103:28.029–28.039.

[plx029-B37] Kirschke S, Bousquet P, Ciais P, Saunois M, Canadell JG, Dlugokencky EJ, Bergamaschi P, Bergmann D, Blake DR, Bruhwiler L, Cameron-Smith P, Castaldi S, Chevallier F, Feng L, Fraser A, Heimann M, Hodson EL, Houweling S, Josse B, Fraser PJ, Krummel PB, Lamarque JF, Langenfelds RL, Le Quere C, Naik V, O'Doherty S, Palmer PI, Pison I, Plummer D, Poulter B, Prinn RG, Rigby M, Ringeval B, Santini M, Schmidt M, Shindell DT, Simpson IJ, Spahni R, Steele LP, Strode SA, Sudo K, Szopa S, van der Werf GR, Voulgarakis A, van Weele M, Weiss RF, Williams JE, Zeng G. 2013 Three decades of global methane sources and sinks. Nature Geoscience 6:813–823.

[plx029-B38] Kosten S, Piñeiro M, de Goede E, de Klein J, Lamers LPM, Ettwig K. 2016 Fate of methane in aquatic systems dominated by free-floating plants. Water Research 104:200–207. 27525583 10.1016/j.watres.2016.07.054

[plx029-B39] Laanbroek HJ. 2010 CH_4_ emission from natural wetlands: interplay between emergent macrophytes and soil microbial processes. A mini-review. Annals of Botany 105:141–153. 19689973 10.1093/aob/mcp201 PMC2794055

[plx029-B40] McCullagh P, Nelder JA. 1989 *Generalized linear models* 2nd ed. *Monographs on Statistics and Applied Probability* 37. Chapman & Hall, London.

[plx029-B41] Minke M, Augustin J, Hagermann U, Joosten H. 2014 Similar methane fluxes measured by transparent and opaque chambers point at belowground connectivity of *Phragmites australis* beyond the chamber footprint. Aquatic Botany 113:63–71.

[plx029-B42] Myhre G, Shindell D, Bréon FM, Collins W, Fuglestvedt J, Huang J, Koch D, Lamarque JF, Lee D, Mendoza B, Nakajima T, Robock A, Stephens G, Takemura T, Zhang H. 2013 Anthropogenic and natural radiative forcing In: Climate Change 2013: The Physical Science Basis. Contribution of Working Group I to the Fifth Assessment Report of the Intergovernmental Panel on Climate Change. Cambridge, New York, UK, USA: Cambridge University Press.

[plx029-B43] Natchimuthu S, Sundgren I, Gålfalk M, Klemedtsson L, Crill P, Danielsson Å, Bastviken D. 2016 Spatio-temporal variability of lake CH_4_ fluxes and its influence on annual whole lake emission estimates. Limnology and Oceanography 61:S13–S26.

[plx029-B44] Natchimuthu S, Wallin MB, Klemedtsson L, Bastviken D. 2017 Spatio-temporal patterns of stream methane and carbon dioxide emissions in a hemiboreal catchment in Southwest Sweden. Scientific Reports 7:39729. 28045092 10.1038/srep39729 PMC5206626

[plx029-B45] Nykänen H, Alm J, Silvola J, Tolonen K, Martikainen PJ. 1998 CH_4_ fluxes on boreal peatlands of different fertility and the effect of long-term experimental lowering of the water table on flux rates. Global Biogeochemical Cycles 12:53–69.

[plx029-B46] R Core Team (2013) *R: A language and environment for statistical computing. R Foundation for Statistical Computing, Vienna, Austria* http://www.R-project.org/ (13 July 2017).

[plx029-B47] Rusak JA, Yan ND, Somers KM, McQueen DJ. 1999 The temporal coherence of zooplankton population abundances in neighboring north-temperate lakes. American Naturalist 153:46–58. 10.1086/303147 29578771

[plx029-B48] Segers R. 1998 Methane production and methane consumption: a review of processes underlying wetland methane fluxes. Biogeochemistry 41:23–51.

[plx029-B49] Sorrell BK, Brix H. 2013 Gas transport and exchange through wetland plant aerenchyma. In: DeLaune RD, Reddy KR, Richardson CJ, Megonigal JP, eds., *Methods in biogeochemistry of wetlands*, SSSA Book Ser. 10. SSSA, Madison, WI. 177–196.

[plx029-B50] Symonds MEV, Moussalli A. 2011 A brief guide to model selection, multimodel inference and model averaging in behavioural ecology using Akaike’s informations criterion. Behavioral Ecology Sociobiology 65:13–21.

[plx029-B51] Tornberg T, Bendix M, Brix H. 1994 Internal gas transport in *Typha latifolia* L. and *Typha angustifolia* L. 2. Convective throughflow pathways and ecological significance. Aquatic Botany 49:91–105.

[plx029-B52] Turetsky MR, Kotowska A, Bubier J, Dise NB, Crill P, Hornibrook ERC, Minkkinen K, Moore TR, Myers-Smith IH, Nykanen H, Olefeldt D, Rinne J, Saarnio S, Shurpali N, Tuittila ES, Waddington JM, White JR, Wickland KP, Wilmking M. 2014 A synthesis of methane emissions from 71 northern, temperate, and subtropical wetlands. Global Change Biology 20:2183–2197. 24777536 10.1111/gcb.12580

[plx029-B53] Van der Nat F-J, Middelburg J, van Meteren D, Wielemakers A. 1998 Diel CH_4_ emission patterns from *Scirpus lacustris* and *Phragmites australis*. Biogeochemistry 41:1–22.

[plx029-B54] Wang ZP, Han XG. 2005 Diurnal variation in CH_4_ emissions in relation to plants and environmental variables in the inner Mongolia marshes. Atmospheric Environment 39:6295–6305.

[plx029-B55] Whiting GJ, Chanton JP. 1996 Control of the diurnal pattern of CH_4_ emission from emergent aquatic macrophytes by gas transport mechanisms. Aquatic Botany 54:237–253.

[plx029-B56] Xing Y, Xie P, Yang H, Ni L, Wang Y, Tang W. 2004 Diel variation of methane fluxes in summer in a eutrophic subtropical lake in China. Journal of Freshwater Ecology 19:639–644.

[plx029-B57] Yavitt BJB, Knapp AK. 1998 Aspects of methane flow from sediment through emergent cattail (*Typha latifolia*) plants. New Phytologist 139:495–503.

[plx029-B58] Yvon-Durocher G, Allen AP, Bastviken D, Conrad R, Gudasz C, St-Pierre A, Thanh-Duc N, del Giorgio PA. 2014 Methane fluxes show consistent temperature dependence across microbial to ecosystem scales. Nature 507:488–491. 24670769 10.1038/nature13164

[plx029-B59] Zuur AL, Ieno EN, Elphick CS. 2010 A protocol for data exploration to avoid common statistical problems. Methods in Ecology and Evolution 1:3–14.

